# Facilitating Ambulatory Electronic Health Record System Implementation: Evidence from a Qualitative Study

**DOI:** 10.1155/2013/629574

**Published:** 2013-10-20

**Authors:** Ann Scheck McAlearney, Cynthia Sieck, Jennifer Hefner, Julie Robbins, Timothy R. Huerta

**Affiliations:** ^1^Department of Family Medicine, College of Medicine, The Ohio State University, 273 Northwood and High Building, 2231 North High Street, Columbus, OH 43201, USA; ^2^Division of Health Services Management and Policy, College of Public Health, The Ohio State University, 250 Cunz Hall, 1841 Neil Ave., Columbus, OH 43210, USA; ^3^Department of Biomedical Informatics, College of Medicine, The Ohio State University, 3190 Graves Hall, 333 W. 10th Avenue, Columbus, OH 43210, USA

## Abstract

*Background*. Ambulatory care practices have increasing interest in leveraging the capabilities of electronic health record (EHR) systems, but little information is available documenting how organizations have successfully implemented these systems. *Objective*. To characterize elements of successful electronic health record (EHR) system implementation and to synthesize the key informants' perspectives about successful implementation practices. *Methods*. Key informant interviews and focus groups were conducted with a purposive sample of individuals from US healthcare organizations identified for their success with ambulatory EHR implementation. Rigorous qualitative data analyses used both deductive and inductive methods. *Results*. Participants identified personal and system-related barriers, at both the individual and organization levels, including poor computer skills, productivity losses, resistance to change, and EHR system failure. Implementation success was reportedly facilitated by careful planning and consistent communication throughout distinct stages of the implementation process. A significant element of successful implementation was an emphasis on optimization, both during “go-live” and, subsequently, when users had more experience with the system. *Conclusion*. Successful EHR implementation requires both detailed planning and clear mechanisms to deal with unforeseen or unintended consequences. Focusing on user buy-in early and including plans for optimization can facilitate greater success.

## 1. Introduction

The US Health Information Technology for Economic and Clinical Health (HITECH) Act of 2009 aimed to reduce healthcare costs, improve quality, and increase patient safety by rewarding providers who adopt and meaningfully use certified electronic health records (EHRs) [1]. As a result, the reach of EHRs within primary care has expanded in recent years, with the expectation that EHR use will eventually be required as health care reforms are implemented [2–6]. In addition, the Patient Protection and Affordable Care Act (ACA) enacted in 2010 provides reimbursements to eligible physicians who meet meaningful use criteria in primary care settings, further supporting providers' efforts to adopt and use EHR systems through the establishment of organizations such as Health Information Technology Regional Extension Centers (HITREC) and Health Information Exchanges (HIE). These legislative efforts occurred concurrently with a documented increase in EHR use in primary care offices from 22% in 2009 to 40% in 2012 [5, 7, 8]. Yet while this expansion is substantial, only 27% of office-based physicians who planned to apply for incentive funding had systems capable of meeting Stage 1 meaningful use objectives, suggesting that having an EHR in place is not the same as having the capacity to “meaningfully use” that EHR [5] in ways that truly improve care quality and increase patient safety.

Resistance to EHR adoption and use is not unique to primary care [9], and researchers have noted a slower pace of adoption because of both organization- and physician-derived concerns [4, 6, 7, 10–14]. Notable organizational concerns include issues related to the significant costs associated with EHR deployment, lack of standardization across environments, and potential decreases in productivity (and therefore income), especially during initial implementation [14–16]. Creating EHR interoperability with other health information technology (HIT) applications is also a significant challenge. The ability to integrate EHRs into existing technologies, such as legacy billing systems that were not originally built to handle laboratory data, can slow adoption. The inherently networked nature of HIT systems has also made their coordinated adoption more complicated than for stand-alone technologies and thus slowed their widespread adoption. Further, the patient care tasks in ambulatory care settings may be quite varied, requiring greater adaptation and customization of the EHR, which can lengthen the implementation phase [17]. 

In contrast, physicians have demonstrated a reluctance to embrace EHR technology because of personal factors (unwillingness to change practice), professional factors (perceived threat to professional autonomy), equity factors (efficiency benefits accrue to the hospital rather than physicians), and technical factors (limitations of the technologies) [3, 11, 15, 18–22]. Peterson et al. [9] found that perceptions about the benefits associated with EHR adoption were different among current users in contrast to intended adopters and that the latter had a particularly convoluted mental model associated with the benefit structure for EHR adoption, suggesting no clear value proposition for EHRs over current systems. This uncertainty has created considerable confusion in the evaluation of the impact of EHR systems, with many organizations choosing to define success by considering technical factors instead of patient-driven outcomes [15].

While there are a number of successful pioneer EHR adopters, the chasm between the early adopters and the early majority is difficult to span [23]. While the potential for benefits is clear, successful adoption of EHR systems in ambulatory care settings faces a significant number of barriers that can and do cause implementations to fail. As a result, there is a reticence to adopt and implement EHR systems that may engender yet another costly EHR implementation failure. Less clear is the pathway through these issues to successful implementation, and, as a result, more analysis of the EHR adoption journey is needed, particularly demonstrating how physician practices and organizations have overcome these barriers and become successful implementers [15, 24–28].

This study was designed to explore physicians' and organizations' perspectives about how adoption and implementation of an electronic health record (EHR) system can be facilitated in ambulatory practice settings. We conducted in-depth qualitative interviews and focus groups to better understand and describe the process through which successful sites implemented their EHR. Our study was guided by the five states of the adoption process outlined by Rogers [29] in his research on the diffusion of innovations. Those stages—knowledge, persuasion, decision, implementation and confirmation—outline a process through which ideas become adopted into practice. In this study, we particularly focused on the fourth and fifth stages of Rogers' model, implementation and confirmation, because it is in these last two stages that an organization deploys the technology, shifting from decision to action and then reassesses its decision to implement. In this paper we characterize elements of EHR implementation processes consistently present in healthcare organizations that were reported to have “best practices” in ambulatory EHR system implementation. We synthesize and summarize informants' perspectives about those exemplary implementation practices in this paper.

## 2. Methods

### 2.1. Population Studied

We interviewed 45 physician and organizational representatives across six organizations that were considered as exemplars for successful ambulatory EHR implementation. The sites were selected using an iterative process. First, we generated a list of potential sites based on information from a variety of sources, including (1) winners of the Healthcare Information Management Systems Society (HIMSS) “Davies Award” which recognizes excellence in EHR implementation; (2) *Hospital and Health Network's* benchmark survey of the “most wired” healthcare organizations; (3) resources and conversations with leading EHR vendors; and (4) recommendations from a “Project Advisory Committee” comprised of health care executives participating in an academic-practice research collaborative that funded our study. From this list, we purposively selected study sites based on a subjective assessment of the strength of their reputation (e.g., evidence from more than one of our information sources), organizational characteristics that would ensure variation across our sample (e.g., geography, organizational size), input from Advisory Committee members regarding learning opportunities related to each site, and practical considerations (e.g., existing contacts at the sites). At each site, we interviewed a range of key informants including information systems directors, organizational leaders, medical directors, clinicians, finance and accounting personnel, clinical staff, and information technology managers, trainers, and staff. 

In addition, we conducted six focus groups comprised of 37 physician providers. These providers included physician residents in training as well as attending physicians and physicians in private practice. Additional information about our study participants is provided in Table 1.

### 2.2. Data Collection

We designed in-depth interview guides comprised of open-ended questions for both the focus groups and key informant interviews. Our techniques conformed to established standards for rigorous qualitative research outlined by others' research [30, 31], including guidelines for in-depth interviews (e.g., [32]) and focus group (e.g., [33]). 

Interviews lasted 30–60 minutes and were recorded and transcribed for later analysis. Focus groups lasted 60–90 minutes and were led by a facilitator with a comoderator available to assist. Focus groups were also recorded and transcribed prior to analysis.

### 2.3. Analyses

Our analytic process used a grounded theory approach [34, 35], combining inductive and deductive methods. In this approach, the research team reviewed and discussed transcripts and preliminary findings throughout the data collection phase. After the interviews and focus groups were completed, the lead project investigator established a coding team. The coding team first identified broad themes that emerged from the interview guides and transcripts and established a preliminary coding dictionary, organizing the data into categories of findings [36]. After the preliminary coding, the team developed a list of codes and coding frame, which we then applied to the qualitative data we had collected. To clarify codes and themes as well as to establish consistency across coders, each team member initially reviewed three common documents and compared results. The team also held regular discussions throughout the coding process to enhance consistency and reach consensus on themes that emerged during analysis. We also conducted a concurrent review of literature on EHR system implementation to allow comparisons with existing data, when appropriate [34]. Our team used the qualitative data analysis software program Atlas.ti (version 6.0) to support detailed coding and more formal exploration patterns and themes within the data [37].

## 3. Results

### 3.1. Perspectives about EHR Implementation Challenges

Study participants were asked direct questions about the EHR implementation process, including about the challenges of implementation. We categorized responses by type, reflecting personal or system-related barriers, at either the provider or organization level. Personal barriers at the provider level included difficulty in changing work patterns, lack of computer skills, and the nearness of retirement; system-related barriers at the provider level involved providers wishing to customize the system, loss of productivity, and loss of ability to document in detail. At the organization level, personal barriers included general resistance to change, lack of perceived value from the EHR system, and perceptions of insufficient support for EHR use. System-related barriers at this organization level involved challenges such as the system going down, the system's limitations, and managing system updates. In Table 2 we present representative comments that were reflective of the personal and organizational barriers respondents perceived to be associated with ambulatory EHR implementation.

### 3.2. Staging the Implementation Process

Using the conceptual framework we established for this exploratory study, we sought to characterize distinct elements of EHR implementation process that could be associated with the Implementation Phase of the Rogers' Innovation-Decision process [29]. Across sites, we identified four phases of the EHR implementation process in these ambulatory care settings: (1) Adoption Decision; (2) Vendor Selection; (3) Implementation Planning; and (4) Implementation “Go-Live.” Below we describe our characterization of each of these discrete phases in greater detail, and in the list below we present comments from our study participants as representative evidence for the classification of these implementation phases.

*Representative Comments about Staging the EHR System Implementation*

1. Making the Ambulatory EHR Adoption Decision
  - “A formal strategic initiative was developed and submitted and required approval for funding since obviously it was huge, the funding dollars that were involved.” ~Physician
  - “It was across the entire system, it was not something that was in any way shape or form limited to simply just the IT department.” ~Executive
2. Vendor Selection and Support
  - “There was more your standard vendor analysis process that went on to figure out what the best product was in relation to what [the health system's] needs were, and so forth.” ~IT Professional
  - “We leveraged a very strong partner, an outsource vendor… They've got great resources, they have very significant experience and knowledge and have done a very nice job for us.” ~Executive
3. Implementation Planning
   
*Readiness Assessment*
    (i) “So what we do then is we evaluate each of the people and we assess them as far as, ok now we've identified each of the people that we think are going to need maybe some extra training versus others. And then it just gives you a feel as far as the doctors. “Do I feel comfortable as far as using an electronic medical record? Do I think an electronic medical record is going to benefit my practice? What are the detriments of a medical record? Do you think it's going to help the communication?” Based on some of those answers we can tell the attitudes and we can tell where we're going to find some challenges.” ~Manager
   
*Structuring a Project Management Process*
    (i) “There was a really aggressive roll-out plan to do it. There were different scenarios: two years, three years, four years, five years.” ~Manager
    (ii) “[On the project plan is] everything you need to do starting from the walkthrough of the hardware, doing the tutorial, getting all the hardware in place. And we have a check off—is this done? Is this done? Turning on interfaces, end user training, any clinical content, end user training, go live. And any follow-up and any process improvements that we do along the way. So yeah, everything.” ~Manager
4. Implementation Go-Live
   
*Workflow Analysis*
    (i) “We were in meetings for every week for hours upon end doing these screen designs and talking through the flow.”~Physician
    (ii) “We really were learning that and she understood the flow and we understood our flow. XX understood the electronic flow. So she would find out how she needed to make the system work for us and knew the system's limitations and so then she'd say, ‘Well you're not going to be able to do that in the system. We need to work within these limitations.' And so we'd have to bring that back to the group and say, ‘Well we can't do that, so how are we going to…?'” ~Manager
   
*Preloading Data*
    (i) “If those charts aren't preloaded, it not only affects the provider, it also affects the nurse, it affects the staff-lab, because lab now is backed up.” ~IT Professionals
    (ii) “As far as best practice goes,…that formula of making sure that there's a lot of preload and that the people know how to use the system through doing the preload…” ~Executive
   
*Mitigation*
    (i) “Part of what we anticipated during the implementation would be a reduction in physician productivity. We gave them a six-week period of time to do that—to go back and build their skill set. Six week period—they were given a guarantee in terms of their capitation during that period of time.” ~Manager
   
*Support*
    (i) “And then just a really good support staff that's going to be able to help them through this process. And I need quality people to be able to send out there. I can't send somebody who's marginal out to a practice and expect to have successful go-live.” ~Manager

#### 3.2.1. Ambulatory EHR Adoption Decision

The first phase of implementation described across all sites involved the decision to adopt an ambulatory EHR system. Part of this decision was the articulation of a clear rationale, vision, and goals for EHR adoption, and these were clearly communicated to key stakeholders early and consistently. Across sites, informants emphasized the importance of early physician buy-in, with several sites noting that, ideally, the physicians themselves should drive the EHR adoption decision as opposed to the health system. Early and strong physician buy-in was deemed critical in order to ensure successful implementation and minimize resistance. During this adoption decision phase, informants also noted that organizations must recognize the significant investment required and that the investment involved a sustained commitment of both financial and nonfinancial resources (e.g., time, commitment to change).

#### 3.2.2. Vendor Selection and Support

The second phase we characterized in the implementation process involved selecting an EHR system vendor for system implementation and ongoing support. Several best practice sites viewed their EHR vendor as an active “partner” in the EHR implementation process, but this was not articulated as a firm requirement. Informants did note, however, that physician users should be an integral part of the vendor selection process to ensure that the product selected could support their clinical needs/preferences.

#### 3.2.3. Implementation Planning

Prior to implementation, successful organizations took a strategic approach to minimize disruption to the organization. Several organizations incorporated a “readiness to change” assessment as part of this planning phase, using structured and formal processes to assess “readiness for implementation” at both the clinic (e.g., current use of IT, clear understanding of change needed) and individual clinician levels (e.g., typing skills). Information from the change readiness assessment informed decision-making and focused support efforts as the implementation progressed (e.g., making a decision to support typing skills training).

Additionally, best practice sites discussed how they used a project management approach to implementation that included several key components: (1) having a designated project leader; (2) establishing clear timeframes and accountabilities; (3) incorporating a systematic approach to feedback and improvement; and (4) ensuring clear and consistent communication. While the strategy was consistent, the personnel charged with leading components varied across sites. In some sites, the health system assigned IT-based project managers to partner with individual practices and clinics. In other sites, individual leaders within the practice were identified to lead the project. Regardless of the model used, informants from all sites highlighted the need to dedicate resources to the EHR implementation.

Further, communication was repeatedly emphasized as critical to success during the Implementation Planning phase. A clear understanding, across all parties about their role(s), the timeframe for implementation, and the expected impact of the new system, was identified. Messaging was also important, for instance, the need to consistently communicate the message that this transition was not one that could be completed “on the side.” By managing expectations and creating a system for constructive dialogue, negative word-of-mouth exchanges that reduce the likelihood of successful implementation could be addressed before they influenced attitudes towards EHR adoption, acceptance, and use. 

#### 3.2.4. Implementation Go-Live

The final phase of implementation representing the switchover to the new system had six subelements: (1) phasing; (2) workflow analysis; (3) training; (4) preloading; (5) mitigation; and (6) support. First, the process of implementation phasing in these sites consisted of determining the pace and scope of the implementation “go-live,” ranging from a “big bang” approach in which the EHR was implemented “overnight” within a site (or across sites), to a more conservative approach in which various functions of the EHR were phased in sequentially. Each approach appeared to have both benefits and drawbacks and we found no approach that was the most successful for phasing the implementation.

Second, as informants across sites are noted, a detailed workflow analysis and redesign were critical in order to ensure that the EHR could be utilized effectively; without this detailed process, organizations reportedly risked transferring bad practices from the paper environment into the electronic world and missed opportunities which the EHR provides to improve practice. Most sites viewed the workflow analysis process as a way to review and improve clinical practice and operations.

Third, while all sites required physicians to participate in a formal training prior to implementation go-live, the most successful trainings were conducted within a few weeks of go-live. Many informants noted that giving physicians the opportunity to “play around” with the system prior to going live was very valuable, but informants also reported they typically found that many physicians were unwilling to dedicate the time to learning about the EHR until it was absolutely necessary. One health system with a very sophisticated training model had evolved their training approach into a “competency-based” model. In this organization, physicians had mandatory e-learning requirements and then had to demonstrate competency with basic EHR functions before they could use the new EHR system for patient interactions (e.g. place an order, document patient history and physical, etc.).

Fourth, although time-consuming, many sites reported that having physicians abstract their own charts and “*pre-load*” data was an important part of the training process. Even if physicians only did a minimal amount of preloading of their clinical data, this hands-on effort appeared to help increase physicians' comfort with the EHR system before going live with the EHR during a patient encounter. This early work with the data provided physicians with exposure to seeing records in electronic form and gave them another opportunity to interact with the system.

Fifth, all sites planned for reduced productivity levels during the go-live period, and reduced productivity was usually expected for 1-2 months. Productivity levels were typically reduced to 25% or so of original levels during an initial go-live phase, and then were bumped up incrementally (e.g., to 50%, then to 75%, etc.) as users gained proficiency with the system. Although most sites reported a return to full productivity, others had reportedly struggled to return to original levels. It was suggested that the link between physicians' compensation and productivity expectations could make a difference. Several sites noted that compensating physicians for training time helped increase participation rates, but not all sites had the financial resources or flexibility to provide this support.

Sixth, support was a critical component of the implementation go-live period. We found that most sites offered on-site IT support (vendor and/or internal IT) during the go-live period (e.g., “red coats” who were available on-site during go-live to answer questions and help troubleshoot on the spot). It was reportedly critical that the on-site support persons have strong technical knowledge of the EHR system as well as clinical knowledge. Informants across sites reported that a capable support team was an essential facilitator of successful implementation and noted that in-person support was particularly wellreceived by physicians anxious about the transition to the EHR.

### 3.3. Emphasizing Optimization as Part of the Postimplementation Process

The final key component of successful EHR implementation involved an emphasis on optimization of EHR system use. Study participants noted two time frames during which optimization could be particularly valuable. First, a focus on optimization immediately after the system went live reportedly minimized frustration at a time when users were on a steep learning curve. Then, once physicians had some experience using the EHR system, a second opportunity arose when users were more engaged in the system and interested in “optimizing” their use of the EHR. Ideally, EHR implementation itself was described as a process of ongoing learning and improvement, and recognizing opportunities for optimization was noted to be an important part of this process.

We categorized four subthemes related to optimization in EHR implementation: (1) acknowledging optimization as a specific component of implementation; (2) supporting optimization of EHR use; (3) EHR user recognition of the value of optimization; and (4) the need to view optimization as an ongoing process. We present representative quotations associated with these four subthemes involving optimization in the list below.

*Emphasizing Optimization in Ambulatory EHR Implementation*

 
*Defining Opportunities for Optimization*
  (i) “When you think about going forward, I hear there [are] human factor things that are going to come up as the implementation is wrapping up. There is the optimization. What are other things that are on the drawing boards for you all going forward?”~Manager
  (ii) “We've actually been doing optimization for, after all the clinics went live, for a little period of time after they went live, maybe 6 to 10 weeks we did a little touch points but now, a lot of them it's a year past go-live and we're still out there doing the optimization.”~IT Professional
 
*Supporting Optimization of EHR Use*
  (i) “The optimization on the clinics side is coming out of the clinic education department. These are half a dozen people—they do the training for some of the doctors.”~Manager
  (ii) “So they're really out there kind of on the cutting edge of looking for more things to use the system for.”~IT Professional
 
*EHR User Recognition of the Value of Optimization*
  (i) “The people that are here now have really embraced it and they're more looking… they love the optimization. They love it…” ~Manager
  (ii) “When you go out there and show them something different it's a big “aha!” moment and they really embrace it immediately because they can see that it's a win.” ~IT Professional
 
*Valuing Optimization as an Ongoing Process*
  (i) “They are bringing back those optimization ideas and we are trying to spread them throughout the clinics as better work processes.” ~Manager

## 4. Discussion

Ford and colleagues recently noted that qualitative studies were needed to better understand HIT implementation issues: “during this transitional phase, systematic qualitative studies would be the most valuable to both policy makers and leaders wishing to explore the management of information systems” [38]. Our study directly addresses this need through the conduct of interviews and focus groups in a rigorous qualitative research design appropriately suited to elucidate the key issues faced by providers and organizations during EHR implementation. This research can serve as the basis for the development of questions for future surveys, as well as for secondary research on larger samples as EHR implementation proliferates. 

Our results support the maxim that accomplishment is detail dependent and success is interpersonal. Specifically, many of the successful implementation strategies we identified were the result of both meticulous planning and clear mechanisms to deal with unforeseen or unintended consequences. Further, the interpersonal issues are crucially important—EHR adoption is a cultural revolution [39] of both new patterns and tools. For instance, as younger physicians are brought into the clinical community, they bring with them an increased familiarity with EHRs. Many medical residents have never used a paper-based health record system. As a result, the medical field is experiencing an evolutionary shift in their expectations for clinical systems in the hospital environment, creating new internal influences on technology innovation.

Research conducted by Lapointe and Rivard [40] suggests that if implementation issues are not addressed promptly, resistance to the system will escalate [40]. Our results indicate that, as the ambulatory practice moves through the implementation process, early resistance relates primarily to the system usability and system features. This must be viewed as a “window of opportunity” [40]. Addressing these issues through system optimization will lessen the likelihood of the resistance becoming politicized.

From a management theory standpoint, this research highlights the complexity of the Implementation Stage of Rogers' Innovation-Decision process and proposes a distinct “optimization” phase of implementation that can enable organizations to progress to the Continuation Stage of the Innovation-Decision. While this characterization shares elements of others' discussions of innovation modification during the adoption stage of the innovation-decision process (e.g., [41]), our explication of the optimization phase as part of the actual implementation phase is novel. 

Finally, productivity hits are to be expected, but they can be planned. There is a learning curve associated with EHR implementations, and the time for this learning must be accommodated. In addition, prior research has shown that incremental implementation strategies are associated with better productivity outcomes [42, 43]. Firms' willingness to innovate despite temporary declines in efficiency is driven by the desire to stay within the institutional norms of their sector [44]. In practice, firms willingly incur lower levels of efficiency to develop or implement innovations [45]. Further still, firms will purposely lower efficiency to increase effectiveness in sectors wherein competition is premised on quality [46] and differentiated care delivery models [47]. Healthcare organizations can take advantage of lessons learned in healthcare and across industries to modify productivity expectations and develop appropriate ways to accommodate the required learning curve associated with EHR implementations. 

### 4.1. Limitations and Suggestions for Future Research

Several limitations to the current research need to be addressed. The first is the small number of hospitals that were contacted for the study. It should be noted, however, that given the small number of ambulatory practices that have been recognized for having fully implemented an EHR system that meets the new federal regulations [5], small studies have to be the norm. Further, the time and energy requirements associated with qualitative studies create significant barriers to studies involving larger sample sizes. Nonetheless, the large number of key informants we interviewed across organizations and the diversity of our interviewees gives us confidence in our findings. In addition, this thorough qualitative study is a crucial first step toward the development of future quantitative studies with expanded sample sizes; studies with larger sample sizes would allow for a systematic examination of the role of potentially important factors such as organizational characteristics and physician employment status and consideration of additional perspectives, including those of nurses and other allied health personnel, that we could not address in this study. 

As meaningful use regulations have incentivized more practices to adopt EHR systems, we expect to see more discussion of the successes and failures of these efforts. It remains to be seen whether these first adopter sites will adopt strategies that are entirely different from strategies used by later sites, for instance, by selecting the alternative that is least costly in the short term in an effort to take advantage of the meaningful use program's phased reward program only to see long-term costs increase as a result of that short sightedness. Alternatively, practice leaders may simply choose to take on the project in one fell swoop and use the single vendor approach. Future qualitative research will be crucial to the exploration of these adoption dynamics.

## 5. Conclusions

As the healthcare system moves toward greater and more meaningful use of EHR systems, individual organizations benefit from understanding not only barriers to implementation but also critical elements of the implementation process. This study examines organizations that have come to the latter two stages of rogers' diffusion theory, and, in many ways, these two stages represent the light at the end of the tunnel for those embarking on or traversing through the initial stages of their adoption journey [48]. The story of these journeys highlights components identified by organizations using exemplary EHR implementation strategies. In so doing, we offer narratives to inform how organizations can facilitate more effective hit implementation and use. Key among these successful strategies are early and frequent input from stakeholders, particularly physicians and other end users, and allowing for periods of optimization of the system during the implementation process. These periods allow users to adjust to the system and then begin to consider how it can be most useful for them. They are viewed by best practice organizations as opportunities rather than draws on productivity. Such an approach can facilitate better adoption and more meaningful application of EHR systems, potentially enabling attainment of the ultimate goals of improved patient care and safety.

## Figures and Tables

**Table 1:** Key informants and focus group participants, by role.

Key informants	Number

Executives	7
Managers	11
IT Professionals	13
Physicians	10
Nurses	4
Total	**45**

Focus group participants	Number

Practicing physicians	20
Physicians in training	17
Total	**37**

**Table 2:** Personal and system-related barriers challenging EHR implementation.

	Personal barriers	System-related barriers
Provider-level barriers	*Difficulty changing work patterns* “Our workflow has to change completely” ~Manager *Lack of computer skills* (i) “Well one of the biggest barriers is lack of PC skills.” ~Manager (ii) “I also have a difficult time typing and not looking at my patients.” ~Physician *Threat of retirement* “They are ready to make the move, they've been thinking about it for a while - and they just end up retiring.” ~IT Professional	*Providers wanting customization* “The providers wanted a lot of customization. Things that maybe the system didn't have the capacity to do. Or they wanted everything customized to their department or to their clinic.” ~IT Professional *Loss of productivity, especially initially* (i) “It slows them down” ~Physician (ii) “There were a significant number of common tests that we ordered that were not programmed before we went live and there were the names of some of the tests were not standard and so led to a significant amount of inefficiency.” ~Physician *Loss of ability to document in detail* “I end up doing very abbreviated sentences and not perhaps, including some of the details because it's too long.” ~Physician

Organization-level barriers	*General resistance to change* “We don't want to do that because we've been doing it this way! We've been doing it this way for ten years!” ~Manager *Value of sharing data not seen* “We haven't yet achieved the value of the sharing of data” ~Physician *IS support not perceived as sufficient* “And they weren't able to respond in real time … The help people had no ability to do anything other than to submit a ticket and then it would go to the programmers and then when they got to it they would put it in.” ~Physician	*System goes down* (i) “When the system goes down for any reason, it's not very nice.” ~Manager (ii) “It's crippling … You can't really even see patients because you don't have anything. You don't have a med list, you don't have a problem list, how do you see a patient?” ~Manager *System has limitations* (i) “The query system for me is slow, it's more cumbersome.” ~Manager (ii) “being in that system for some of our patients and then having to go to (another system) for some of our patients.” ~Executive *Challenges managing system updates* “There's so much new to add. We basically, when we get an upgrade, we have to sit down and decide how much of it we can put in because even if we can test it all, we can't train everybody on all of it.” ~Executive
